# How Neutrophils Shape Adaptive Immune Responses

**DOI:** 10.3389/fimmu.2015.00471

**Published:** 2015-09-14

**Authors:** Pieter H. C. Leliefeld, Leo Koenderman, Janesh Pillay

**Affiliations:** ^1^Department of Surgery, University Medical Center Utrecht, Utrecht, Netherlands; ^2^Laboratory of Translational Immunology, University Medical Center Utrecht, Utrecht, Netherlands; ^3^Department of Respiratory Medicine, University Medical Center Utrecht, Utrecht, Netherlands; ^4^Department of Anaesthesiology and Critical Care, University Medical Center Utrecht, Utrecht, Netherlands

**Keywords:** inflammation, immune-regulation, neutrophil, myeloid-derived suppressor cells, immune-paralysis, neutrophil phenotypes, T-cells

## Abstract

Neutrophils are classically considered as cells pivotal for the first line of defense against invading pathogens. In recent years, evidence has accumulated that they are also important in the orchestration of adaptive immunity. Neutrophils rapidly migrate in high numbers to sites of inflammation (e.g., infection, tissue damage, and cancer) and are subsequently able to migrate to draining lymph nodes (LNs). Both at the site of inflammation as well as in the LNs, neutrophils can engage with lymphocytes and antigen-presenting cells. This crosstalk occurs either directly via cell–cell contact or via mediators, such as proteases, cytokines, and radical oxygen species. In this review, we will discuss the current knowledge regarding locations and mechanisms of interaction between neutrophils and lymphocytes in the context of homeostasis and various pathological conditions. In addition, we will highlight the complexity of the microenvironment that is involved in the generation of suppressive or stimulatory neutrophil phenotypes.

## Introduction

Neutrophils are particularly known for their potent anti-microbial functions (1). This notion is enforced by various congenital neutrophil deficiencies, which show marked clinical phenotypes characterized by enhanced susceptibility to bacterial and fungal infections (2, 3). Infections, sterile inflammation, and other non-chronic challenges to the immune system are characterized by a rapid influx of neutrophils into the affected tissue (4). These neutrophils respond to chemo-attractants and adhesion molecules expressed on endothelial cells, and their main function is to clear infections and/or debris. In addition, they influence inflammatory responses through interactions with various cells of the immune system, such as antigen-presenting cells (APCs) and lymphocytes (5, 6). This has been observed in both murine models and in *ex vivo* studies with isolated cells from humans. Although neutrophils have long been considered to be composed of a homogenous population, an increasing body of literature supports the presence of multiple neutrophil phenotypes in cancer and inflammation (7–10). This heterogeneity can be induced by specific differentiation programs in the bone marrow or orchestrated by extracellular signals derived from inflammatory tissue (e.g., cytokines, bioactive lipids, or chemokines) (11, 12). The contribution of distinct neutrophil populations to immune suppression has not been resolved. In addition, in murine models and some human studies, clear distinctions were suggested between neutrophils and granulocytic myeloid-derived suppressor cells (G-MDSCs). These issues have been reviewed in detail (13, 14). This review will focus on the location and relevant diseases in which both (suppressive) neutrophils and G-MDSCs modulate adaptive immune responses and the mechanisms behind this process.

## Location of the Interaction between Neutrophils and Lymphocytes

### Site of inflammation – bystander response

The early phase of infection is characterized by an influx of neutrophils and monocytes, which precedes the development of an antigen-specific response. Simultaneously, small numbers of T-cells are recruited into the infected tissue. Some of these T-cells can be activated and proliferate ‘‘in situ’’ in response to antigen presentation by myeloid cells (15). In addition, inflammatory cytokines cause proliferation and activation of non-specific T-cells in the profoundly pro-inflammatory microenvironment. This process has been coined the ‘‘bystander response’’ and was first seen in viral infections (16). It has recently been suggested that this bystander response contributes to early pathogen control in mice by enabling bystander memory T-cells to recognize and eliminate micro-organisms, such as *Listeria monocytogenes* infected cells in a NKG2D-dependent manner (17).

It is conceivable that a large and uncontrolled bystander response might predispose for auto-immunity and self-reactivity through the proliferation of self-reactive T-cells (18). It is tempting to speculate that neutrophils are involved to limit and control this bystander T-cell response as the timing of massive neutrophil tissue infiltration and the bystander response coincide.

### Lymph nodes and primary lymphatic tissue

Neutrophils are found both in LNs and spleen particularly under inflammatory conditions (19–24). Dynamic imaging studies have shown that neutrophils are recruited to and form swarms in infected LNs in mice (25, 26). In addition, neutrophil migration to afferent LNs in response to tissue inflammation has been shown in various murine models (19–24).

**Figure 1:**
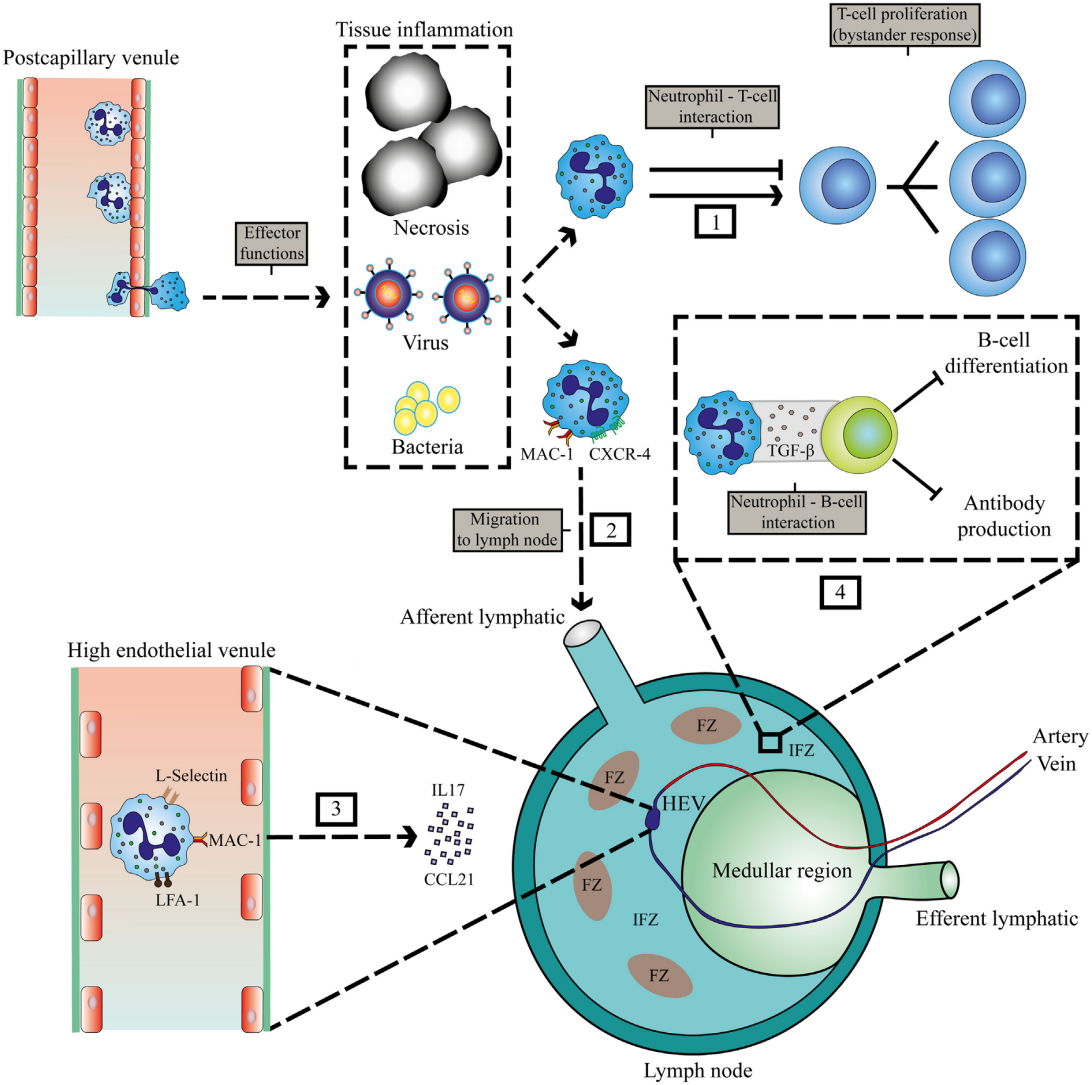
**Localization of interaction between neutrophils and lymphocytes at sites of inflammation and in lymphoid tissue**. Invasion of pathogens or inflammation due to necrosis and cancer leads to extravasation of neutrophils. (1) Interaction of neutrophils with T-cells in the peripheral tissue. (2) MAC-1 and CXCR4-dependent migration of neutrophils to LNs via afferent lymphatics during inflammation (22). (3) IL17 and CCL21-mediated migration of neutrophils via HEVs to the LN that requires MAC-1, LFA-1, and l-selectin (19, 20). (4) Inhibition of humoral responses by neutrophils in the IFZ and medullar region (23). HEV, high endothelial venule; FZ, follicular zone; IFZ, interfollicular zone.

There are two possible routes for neutrophils to enter LNs, via blood vessels or via afferent lymphatics (Figure 1). The first route requires exiting the circulation via high endothelial venules (HEVs). This mechanism is controversial, as human neutrophils seem to lack the expression of CCR7, a receptor for CCL21 and required for lymphocyte exiting through HEVs (27). Nonetheless, it has been shown in a murine model of ovalbumin-induced inflammation that neutrophil homing to LNs via the HEV takes place and requires integrins αMβ2 (MAC-1), αLβ2 (LFA-1), and l- and P-selectin (19). In LN-draining inflammatory tissue, additional chemokines and cytokines could orchestrate the attraction of neutrophils via HEVs. This has also been shown in tumor-draining LNs, when the tumor was subjected to photodynamic therapy. This treatment induces additional sterile inflammation. In this model, neutrophils are recruited to tumor-draining LNs via the HEV in an IL-17-dependent manner (20).

Neutrophil migration to LNs via afferent lymphatics has been observed in various murine models of infections, vaccinations, and cancer and seems to depend on MAC-1 and CXCR4 expression on neutrophils (19, 22) (see Figure 1). The area in the LN that is occupied by neutrophils will determine which cells they encounter, and how they can influence subsequent immune responses. Neutrophils have been reported to occupy the medullary region and interfollicular zone (23). Neutrophils migrate to these areas in the LN during infection with *Staphylococcus aureus*. There they exhibit short- and long-term interactions with B-cells, thereby inhibiting production of antibodies, and thus humoral responses (23). Furthermore, neutrophil B-cell interactions have also been observed in primary lymphoid organs in various mouse models. In the marginal zone of the spleen, neutrophils were observed to contribute to antibody production and class switching by activating B-cells by producing BAFF, APRIL, and IL-21 (24). In this study, evidence was provided for the existence of a similar population of neutrophils that modulate B-cell responses in humans. However, this remains controversial as in a subsequent study, no splenic neutrophil–B cell interactions could be observed in humans (28). It seems firmly established, at least in various murine models that neutrophils enter primary and secondary lymphatic sites during the immune response evoked by various inflammatory stimuli. Apart from regulating immunity in inflamed tissue, they may play a role in regulating immune responses at these privileged immune sites (see below).

## The Role of Neutrophils in Controlling Immune Responses Evoked by Bacterial and Viral Infections, Sterile Inflammation and Cancer

### Viral infections

Acute viral infections, such as influenza, are ideal models to study cellular kinetics during the immune responses and the putative modulating effects of neutrophils hereon. Influenza and RSV infections are characterized by an early large influx of neutrophils in the lung tissue followed several days later by a virus specific CD8^+^ T-cell response (29–31). Neutrophils might facilitate the development of this antigen-specific response as they are able to serve as APCs in influenza infection in mice (31, 32). Such antigen presentation by influenza-infected neutrophils has been demonstrated and was found to be mediated by MHC-I and co-stimulatory molecules CD80 and CD86, which leads to induction and activation of anti-viral responses of CD8^+^ T-cells (32). On the other hand, it is tempting to speculate that neutrophils may also inhibit T-cell responses in viral infections by inhibiting T-cell proliferation and inducing T-cell apoptosis. The mechanisms of this suppression will be discussed below and involve reactive oxygen species (ROS), arginase-I (ARG), and PD-L1. This has also been found in other inflammatory scenarios (33–35). The role of immune suppression by neutrophils *in vivo* in murine models of viral infections has not been adequately experimentally verified, but may be deduced from the fact that pathology in mice is T-cell dependent and that depleting neutrophils often results in an exaggerated response and pathology (31, 36). In chronic viral infections, such as human hepatitis B virus (HBV), it has recently been shown that recruitment of neutrophils to the liver limits immune pathology through inhibiting bystander and HBV specific T-cells in an arginase-dependent way, thus protecting the host from immune-mediated damage (37). These neutrophils were isolated from the PBMC fraction and were termed G-MDSC (13).

### Bacterial infections

As in viral infections, bacterial infections are associated with a large recruitment of neutrophils. The pivotal difference with viral infections is that phagocytosis and killing of bacterial targets by phagocytes is the principle mechanism of pathogen eradication (1). Ineffective killing of phagocytosed bacteria results in intracellular (phagosomal) survival and can lead to pathogen shuttling to distant sites and LNs (38, 39). Recently, it has been shown that neutrophils from mice infected with *S. aureus* migrated to the draining LNs and limited humoral responses through direct cellular interactions with B-cells. These direct cellular interactions were also found for neutrophil–T-cell interactions in humans (40). Kamenyeva et al. suggested that suppression of antibody production by neutrophils *ex vivo* was dependent on TGF-β (23). However, the contribution of the reduced humoral response to pathogen load was not assessed.

As mentioned above, in the early course of infection, large numbers of neutrophils are recruited to the affected tissue where modulation of T-cell responses most likely occurs with early recruited T-cells. These early lymphocytes mainly belong to the family of γδ-T-cells (41). These γδ-T-cells are thought to play a role in early pathogen clearance through production of cytokines and their crosstalk with innate immune cells (41). Neutrophils play an important role in the initiation of these γδ-T-cell responses. Phagocytosis of bacteria enables neutrophils to activate γδ-T-cells and induce their proliferation (42). This is dependent on (1) the microbial metabolite (E)-4-hydroxy-3-methyl-but-2-enyl pyrophosphate (HMB-PP), which neutrophils release after phagocytosis of bacteria and (2) the presence of monocytes for cellular contact-induced activation (42). However, other human studies have revealed the ability of neutrophils to suppress γδ-T-cells activation, possibly providing a negative-feedback mechanism (43, 44).

The early instruction of γδ-T-cells in humans by neutrophils parallels the role of these innate immune cells in the early instruction of T-cell responses in mice. In a murine model of *Legionella pneumophila*, neutrophils from pulmonary tissue are pivotal for the development of a TH1 response. In this model which resembles human disease, neutrophils were depleted by neutrophil-specific antibody Ly-6G, and this led to more TH2 skewing and more disease (45).

T-cell instruction, activation, and proliferation mostly require antigen-presenting cells, such as dendritic cells, B-cells, and macrophages. Neutrophils have been shown to both negatively and positively affect antigen presentation by these APCs under different conditions. This has extensively been reviewed previously (46, 47). Neutrophils may simply affect the amount of available antigen by phagocytosis, and thus limit antigen presentation by professional APCs (48). Alternatively, neutrophils might function as APCs themselves (49, 50). This possibility is supported by several studies showing the expression of MHCII and co-stimulatory molecules on neutrophils under different clinical conditions (51–53).

### Disseminated bacterial infections (sepsis)

Severe bacterial infections can result in systemic dissemination of bacteria that can lead to severe clinical conditions, such as sepsis and septic shock. These conditions are characterized by severe systemic inflammation, which can result in severe inflammatory damage to the host when not properly controlled. Immune inhibitory mechanisms have evolved in order to prevent this exaggerated inflammatory response (54). The specific role of neutrophils in this immune suppression has not been adequately studied. This is a challenging research question as depletion or inhibition of neutrophil functions with the purpose of studying their anti-inflammatory role has profound impact on bacterial clearance. Identification of suppressive mechanisms that do not influence pathogen clearance and neutrophil-specific murine knockout models may aid in answering this question.

In humans, evidence has accumulated that neutrophils might contribute to the immune suppression seen in sepsis. Neutrophils in septic-shock patients express ARG and suppress T-cell functions, probably through depletion of l-arginine as detailed below (55). Immune suppression in sepsis can be at least in part attributed to the PD-1/PD-L1 axis that is involved in control of apoptosis in T-cells (56). Interestingly, expression of PD-L1 on tissue neutrophils has also been shown during chronic inflammation (57). The expression of PD-L1 on human neutrophils was found to be induced by the TH1 cytokine, interferon-γ, *in vitro* (35).

### Sterile inflammation/vaccination

Neutrophils also play a role in the fine tuning of inflammation under sterile conditions. Many studies have been performed in ovalbumine (OVA)-induced immune responses in murine models. The OVA models are used as vaccination and allergy models and are useful to study the development of adaptive immune responses. The role of neutrophils in the OVA model follows the above-described findings in microbial models. The cells seem to effectively cross-prime CD8^+^ T-cells in an MHCI-dependent manner (58). They function as APCs or influence the capacity to present antigens by professional APCs (59, 60). For instance, dendritic cells have been shown to take up antigens acquired from phagocytosed apoptotic neutrophils (61).

These examples show that neutrophils can increase antigen presentation as APC or by delivering antigen to APCs. On the other hand, there are reports that neutrophils decrease the level of antigen presentation by APCs through an unknown mechanism during brief cellular interactions (48). These findings show that it is difficult to predict in which circumstances neutrophils stimulate or suppress antigen presentation even when very similar and well-controlled models are used.

### Cancer

There is a large body of literature, which shows a heterogeneous population of myeloid cells characterized by their potential to inhibit adaptive immunity, and thus anti-tumor immune responses (14). These myeloid-derived suppressor cells consist of mononuclear cells and neutrophils in different stages of maturation. G-MDSCs facilitate tumor growth in various murine models through suppression of CD8^+^ responses and production of cytokines (62–64). In addition, in human cancer patients, G-MDSCs and suppressive neutrophils are isolated from the peripheral blood (65, 66). Although the distinction between neutrophils and G-MDSCs is not clear, the modulating role of these cells in the immune responses induced by tumors has become an accepted paradigm, and is extensively reviewed elsewhere (13, 67). Neutrophils are involved in both pro- or anti-tumor immune responses. Importantly, they have recently been shown to promote metastasis (7, 68). Their anti-tumor effects are mediated by their direct antibody-dependent cytotoxicity and their production of pro-inflammatory cytokines near and inside the tumor (69). These properties will not be discussed in this short review.

The pro-tumor effects of neutrophils are mediated by different mechanisms. First, neutrophils play an essential role in angiogenesis through expression of matrix metallo-proteases, such as MMP9 (70, 71). Second, in multiple murine models they inhibit anti-tumor CD8^+^ T-cell responses through mechanisms described below. The suppression of anti-tumor T-cell responses by neutrophils was recently shown to be pivotal in tumor metastasis in a murine model of breast cancer (7). In this study, γδ-T-cells facilitated neutrophil recruitment to the tumor via an IL-17 and G-CSF mediated pathway. The microenvironmental cues for the switch in neutrophil phenotype from pro- to ­anti-tumor are slowly being unraveled. In a model of lung cancer, TGF-β induces or recruits neutrophils with a pro-tumor phenotype (termed N1), whereas blocking TGF-β induces an anti-tumor neutrophil phenotype (termed N2) (11).

## Mechanisms of T-Cell Suppression by Neutrophils and G-MDSCs

**Figure 2:**
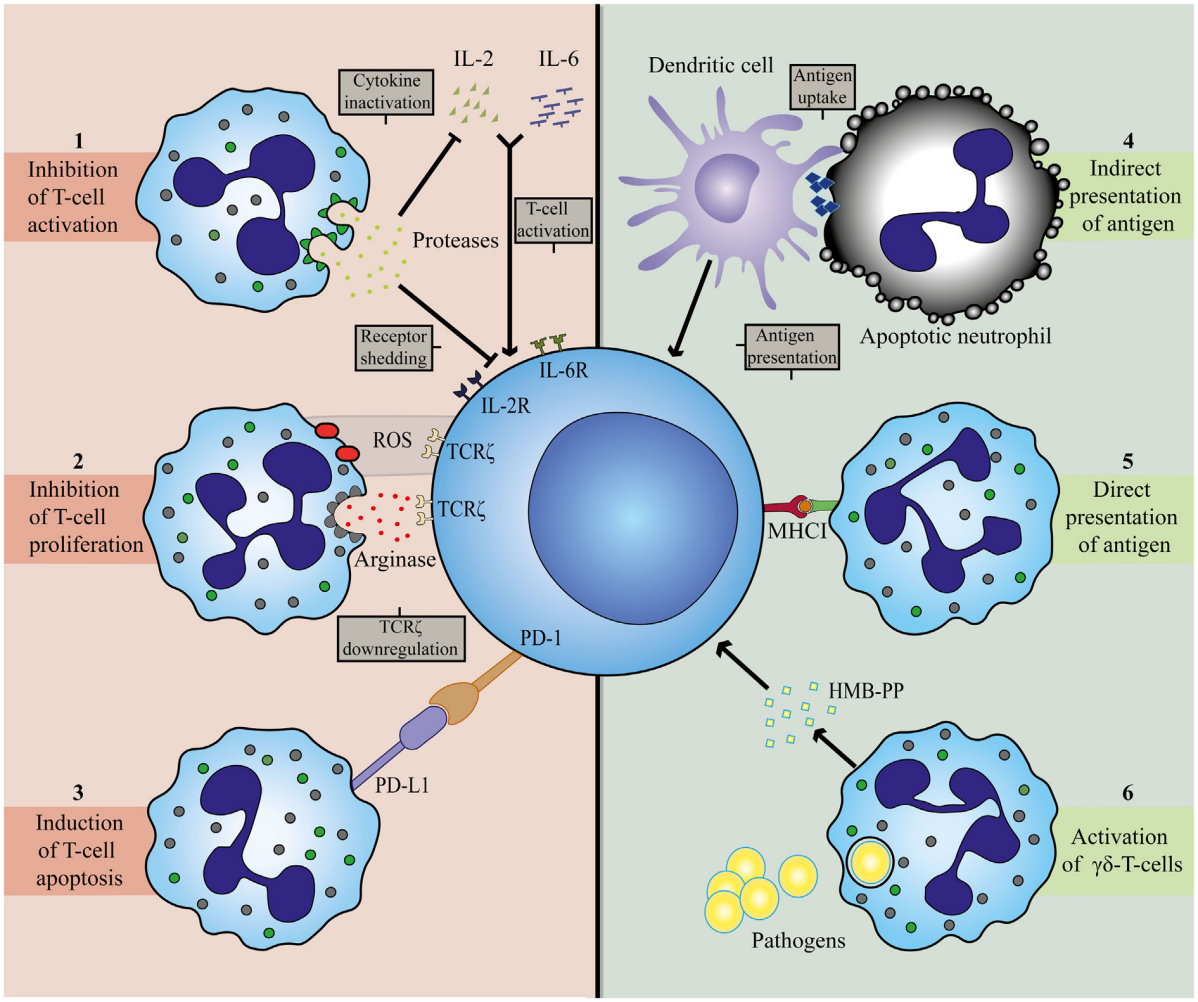
**Mechanisms involved in T-cell inhibition (left panel) and activation (right panel) by neutrophils**. Neutrophils can establish T-cell inhibition by (1) degranulation of granular constituents. The serine proteases elastase and cathepsin G inactivate T-cell stimulating cytokines, IL-2 and IL-6, and catalyze shedding of cytokine receptors for IL-2 and IL-6 on T-cells (72, 73). (2) Production of ROS and release of arginase. Both agents can result in downregulation of TCRζ on T-cells, thereby arresting the cell in the G0-G1 phase (40, 74–78). (3) Expression of PD-L1. Upregulation of this ligand is associated with interferon-dependent PD1-mediated T-cell apoptosis (35, 56). T-cell activation by neutrophils is attained by (4) indirect antigen presentation. Dendritic cells take up antigens from apoptotic neutrophils and serve as APC for T-cells (61). (5) Direct antigen presentation. Neutrophils posses the capacity to cross-prime CD8^+^ T-cells directly in a MHCI-dependent manner (32). (6) Release of microbial metabolites (HMB-PP). Neutrophils release bacterial products after ingestion to activate γδ-T-cells (42).

Despite the evidence that neutrophils can stimulate T-cell responses, most studies point toward a direct suppressive role of these cells on different T-cell responses in various disease models as described above. The mechanisms of suppression have been reviewed in detail elsewhere and are summarized in Figure 2 (13). Most of the mechanisms that neutrophils employ to suppress T-cell functions are closely related to their anti-microbial functions, i.e., the same or similar mediators are used. Two of the most frequently reported mechanisms are via ARG and ROS.

ARG is found in the gelatinase containing granules of neutrophils and is thought to contribute to antifungal immunity trough depletion of l-arginine (79, 80). Depletion of l-arginine also results in a cell cycle arrest in activated T-cells in the G0-G1 phase, which limits T-cell proliferation (74). This is thought to occur through downregulation of TCRζ (75, 76). It seems that the expression of TCRζ requires l-arginine for adequate expression and functionality (81). In addition, l-arginine is required for dephosphorylation of cofilin. Cofilin is pivotal for the stability of the immunological synapse. Therefore, depletion of l-arginine by ARG impairs the formation and stability of an immunological synapse, which is required for T-cell activation and proliferation (82, 83).

ROS are an intricate part of neutrophil anti-microbial defense and the lack of ability to produce ROS as, seen in chronic granulomatous disease, is characterized by severe infections (84–86). One of the products of NADPH oxidase activation is hydrogenperoxide (H_2_O_2_). H_2_O_2_ can suppress T-cell proliferation and activation through various mechanisms. It induces apoptosis, decreases Nf-κB activation, and downregulates TCRζ (77, 78). In addition, T-cell suppression by ROS is also accompanied by the oxidation of cofilin (77, 87). Cofilin can, therefore, be influenced by both ARG and ROS: both mechanisms being employed by neutrophils. Therefore, the T-cell suppression by disruption of cofilin might prove to be a useful therapeutic target.

Interestingly, regulatory T-cells are resistant to oxidative stress (88). This suggests that regulatory T-cells are less sensitive to suppression than other T-cells, thus enhancing the overall suppressive effect of H_2_O_2_.

Suppression of T-cell activation and proliferation requires high concentrations of H_2_O_2_ (33, 87). This amount of ROS might only be reached in inflammatory tissue with massive neutrophil influx. A more elegant way of suppression via H_2_O_2_ is through cell-cell contact between neutrophils and T-cells. Such a direct mechanism for delivery of ROS in an immunological synapse has been identified (29). Here, neutrophil-T-cell contacts were mediated by MAC-1.

Another important suppressive pathway requires similar cell-cell contacts: inhibition via T-cell PD-1 by PD-L1, a potent inducer of apoptosis in T-cells 1, expressed on neutrophils during sepsis (25). The underlying mechanism of PD-L1 expression is an interferon dependent process (35). The PD-1/PD-L1 axis is thought to be an important mechanism in the immune suppression found in sepsis patients by inducing lymphocyte apoptosis and monocyte dysfunction (56). Blocking this axis after the induction of sepsis by administering a PD-1 blocking antibody improves survival in mice by increasing pathogen clearance (89). This suppressive mechanism might be protective in tissues with severe inflammatory infiltrates, but may be detrimental as immune suppression aggravates sepsis. At this moment, one can only speculate regarding the role of PD-L1 on neutrophils in this immune suppressed state.

Finally, neutrophils can modulate T-cells by degranulating granular constituents, such as neutrophil elastase. These proteases are able to cleave and inactivate essential cytokines, such as IL-2 and receptors, such as the IL-2 and IL-6 receptor on T-cells (72, 73).

## Conclusion

The studies mentioned in this review have led to the consensus that neutrophils are capable of modulating adaptive immune responses through interactions with T- and B-cells and possibly APCs. The mechanistic studies in mice have been corroborated with human *ex vivo* data. These studies show that neutrophils are capable of directly interacting with lymphocytes and modulating their responses at local sites of inflammation as well as in draining LNs. One of the key remaining issues is the question whether human neutrophils show functional plasticity as has been suggested by us and others (13, 90). This plasticity can occur at different levels: (1) the existence of functional subsets, which are intrinsically different or (2) the transdifferentiation into suppressive neutrophils or even into an APC type of hybrid cell (90–92). The microenvironmental cues mediating the switch from classical neutrophils to suppressive neutrophils have barely been studied although TGF-β seems to play an important role in microbial and tumor models (11, 23).

In conclusion, murine and human studies to date show that neutrophils are potent modulators of immunity. The first step of establishing a strategy to target immune modulatory neutrophils without influencing their essential anti-microbial functions is finding relevant human diseases in which this modulation plays a pivotal role (93). The unraveling of microenvironmental cues mediating the recruitment of and/or “switching” into suppressive neutrophils in such diseases is essential in understanding and targeting of this pathway.

## Conflict of Interest Statement

The authors declare that the research was conducted in the absence of any commercial or financial relationships that could be construed as a potential conflict of interest.
